# Abstracts from German Journals

**Published:** 1872-08

**Authors:** Ch. Raushenberg

**Affiliations:** Atlanta, Georgia


### ABSTRACTS FROM GERMAN JOURNALS.

BY CH. RAUSHENBERG, M.D., ATLANTA, GEORGIA.

*ON SOME GENERAL PHENOMENA OCCURRING AFTER EXTENSIVE BURNS OF THE SKIN. By Dr. FREDERICK FRANK, of Berlin. (Virchow's Archiv.)*

This article forms a valuable addition to the knowledge of death as it is caused by burns. It is based upon the results of a series of experiments made at the Physiological Laboratory of Berlin by the author, with the assistance of Prof. I. Rosenthal, and takes cognizance principally of those cases where extensive burns were survived one or more days. It furnishes a striking illustration of the fact, that the most careful physiological analysis of pathological conditions, by the logical interpretation of the results of judicious experiments *alone*, can furnish a rational, and therefore safe and truly practical, basis for the conscientious treatment of diseases.

Earlier authors*, governed by the results of numerous experiments demonstrating that even larger mammalia, when the surfaces of their bodies were covered by an imperspirable coating—for instance, varnish—would soon die under a considerable decrease of temperature, were inclined to bring this fact in connection with death caused by extensive burns, and to attribute death, in both conditions, to a peculiar poisoning of the blood, caused by the retention of some deleterious substance in consequence of the interrupted functional action of the skin.

Henle†, Donders‡ and Kühne|| expressed their doubts as to the correctness of this opinion; and Laskewitsch§, the last one who repeated these varnishing experiments, came to the conclusion, that death produced in this manner, apparently by an interruption of the functions of the skin, with the pathological

*Pitha Billroth's Chirurgie, Vol. 1, p. 2.

† Allgemeine Pathologie, Vol. 2, p. 238.

‡ Physiologie des Menschen, p. 458.

|| Lehrbuch der Physiologischen Chemie, 1868, p. 440.

§ Archiv. für Anatomie und Physiologie, von Reichert und Bois-Raymond, 1866, p. 61.

consequences following it, was not the result of the retention of a noxious substance in the blood, but of a morbid decrease of the bodily temperature, caused by dilatation of the cutaneous capillaries.

Dr. F. ~~Frank~~ endeavors to solve the question whether the opinion of Laskewitch, in relation to the causes of death of varnished animals, apply also to death caused by extensive burns of the skin, and whether it can be demonstrated by experiments on animals that these alleged causes are sufficient to explain the death of a large number of these animals.

In conformity with the advice of Claude Bernard* and L. Uterhard†, he preceded the inhalation of chloroform by one subcutaneous injection of morphine, thus producing profound narcosis. Rabbits and dogs, shaved clean all over the surface, were, after the temperature of the body reduced by the shaving had returned to the normal degree, scalded with boiling water, or otherwise subjected to the influence of burning substances. *The most marked symptom, following this lesion invariably in all cases, was a gradual but uninterrupted sinking of the temperature of the body*, which, as clearly established by experiment, was in no degree owing to the narcotized condition of the animals. The temperature was carefully measured in the rectum, and the diminution of it occurred very fast immediately after the infliction of the injury, and afterward continued on gradually until death. It often decreased to 30° Centigrade (86° Fahrenheit) in the first hour after scalding the animals, and when nothing was done to counteract this decrease of temperature, the last respiration of the animal was often noticed at about 19° Centigrade (66.2° Fahrenheit.) The heart continued to beat for a short time afterward; but an increase of the temperature shortly before or after death, which occurred thirteen hours after burning the animal, was never observed.

Do the same phenomena occur in severely-burned human beings? The coldness of burned individuals has frequently been noticed and mentioned by writers, but systematic measurements of the temperature have not yet been made. One

*Bulletin de Therapeutique. †Deutsche Klinische.

case mentioned by Billroth presents an instructive fact. It is that of a man thirty-seven years of age, who, after an extensive burn, producing all the different degrees of this lesion, and without complaining of any pain, showed, two hours after the injury had been inflicted, a temperature of 33° Centigrade (59.4° Fahrenheit.)

The dimensions of the JOURNAL, for which this abstract is prepared, will not permit us to follow Dr. ~~Frank~~ Falk into the details of his interesting experiments on the causes of this diminution of the temperature of the body of scalded animals, and its pathological consequences; and, therefore, we confine ourselves to a reproduction of the most important and most characteristic features contained in the results of his investigations.

He shows conclusively, that neither a suspension of the functions of the skin nor a retention of deleterious substances in the blood can be the cause of the sudden diminution of the temperature of the body of varnished or scalded animals. His experiments lead to the conclusion, that, as a suspension of the functions of the glandular system of the skin could only come into consideration in very deep burns, with destruction of the skin, the sudden suspension of the insensible perspiration alone, without lesion of the skin, is not followed by such a diminution of temperature, or any other unpleasant consequences; that the faculty of the cutis, of absorbing gases or fluids, or excreting foreign substances through its pores or glands, is certainly not diminished, but the first-mentioned frequently increased in a cutis deprived of its epidermis by the effects of a superficial burn.

The correctness of Billroth's theory, that the pathological changes following extensive burns depend upon a poisoning of the blood by the retention of ammonia, is considerably doubted, as traces of free ammonia and carbonate of ammonia have been found by Gerlach, Schottin, and Laskewitsch, on the skin of animals in health, and are the product of a decomposition of different nitrogenous substances, principally epithelial formations, such as hair, nails and epidermis; and that the quantity of ammonia furnished directly by the body is entirely too small, and its effect on the nervous system too plainly that of excitation, and not of depression, as we find it in extensive burns of the human body*.

He arrives at the conclusion, that the diminution of the temperature in the lesion under consideration does not depend upon chemical changes of the blood, but principally upon the physical action of the cutis after burns, and that *the increased loss of caloric through the skin is its principal cause.*

He considers that the physical effect of a temperature far above that of the blood on the skin, when hot water or other substances of a high grade of heat come in contact with it, causes *a dilatation of the cutaneous blood-vessels; hence, hyperæmia of the cutis, and increased radiation and loss of caloric—falling off in the temperature of the body,* under certain circumstances, to as low a degree as that which causes freezing of the body.

His observations, corresponding with those of C. Hastings and O. Weber, on cold-blooded animals and mammalia, relative to the microscopic and histological changes in blood-vessels caused by the effect of a high temperature, prove—

1. That a very marked but gradual dilatation of the arteries, veins and capillaries constitute the main feature of that effect.

2. That this effect is not dependent upon a paralysis of the vaso-motor nerves, or a rigor of the smooth fibres of the vascular walls, caused by the abnormal temperature, but upon a diminution of the elasticity of the walls of the blood-vessels, and of the surrounding tissues generally, by the mere physical effect of heat, in the same way in which that of the metals, for instance, is diminished by heat—so that the pressure of the blood enlarges the volume of the vessels.

The channels of the circulation are thus widened, the quantity of the blood circulating in the affected vessels increased, and, as the larger vessels supplying the affected region are not dilated, the dilatation of the latter leads to a local retardation—perhaps, finally, to a suspension of the motion of the blood-current.

How this increased quantity of blood and diminished velocity of its motion must necessarily give rise to an abnormally in-

* Archiv. für Physiologische Heilkunde, Vol. 1, 1852, p. 73.

creased loss of caloric, can be still better understood if it is taken into consideration, that, according to the corresponding calculations of Helmholtz and Barral, over seventy per cent. of the caloric produced in the body is, in the normal condition, expended through the surface of the body, and that this loss of caloric in burns where the cutis is often deprived of its epidermis, is necessarily increased by increased evaporation. The paradox-sounding assertion, that burned individuals often die from the effects of too great a diminution of their bodily temperature, is nevertheless correct*.

The cause of death in such cases is principally to be found in the fact, that this gradual diminution of the temperature of the blood is followed by a corresponding decrease of the muscular action of the heart, and also, in a less degree, by a corresponding paralysis of the central nervous system.

Upon the basis of these apparently very theoretical experiments, and their results, Dr. ~~Frank~~ makes several very practical suggestions in relation to a rational treatment of extensive burns of the skin. The principal indications to be fulfilled in these cases are—*to counteract the fatal sinking of the bodily temperature, and to elevate it and maintain it as near the normal degree as possible, and to sustain the muscular power of the heart, and to prevent paralysis of the nerve centers.*

The usual treatment of applying different ointments, salves, etc., to the burned surface, whether they are used with that intention or not, accomplish the first object, to some extent, by furnishing an artificial epidermis, and by diminishing evaporation. Wrapping the whole body in cotton wadding answers a similar purpose; but the judicious use of warm baths—which Beaupré† has used in the last century, with the most gratifying effect, in two very severe cases, and which have also been

*It appears that, with this more or less extensive retardation or suspension of the motion of the blood current, and its effect upon the heart and general circulation, a diminution of the metamorphosis and production of caloric in the tissues would step in as an additional factor in maintaining and increasing the low bodily temperature first initiated by increased loss of caloric from the dilated blood-vessels.—Translator.

†James Bird: Lectures on Military Medicine and Surgery—Lecture IV., Lancet, 1835, 2, p. 489.

warmly recommended by Hebra*—furnish, undoubtedly, the most efficient and the most acceptable remedy to elevate and control the bodily temperature, in relation to time and degree, exactly to suit the circumstances of each case. Billroth's interesting statement, that he succeeded, by full baths of several hours' duration, in elevating the temperature, in the case alluded to above, from 33° to 37° Centigrade (91.4° to 98.6° Fahrenheit), furnishes proof to that effect. Valentine†, who first established the fact that animals appparently without animation, in consequence of the effects of varnishing their bodies, could be revived by artificial warming, proposes to establish this in men by heating the air of sick chambers, without charging it at the same time with watery vapors or noxious products of combustion, on the principle that air is not as good a conductor of heat as water.

The general oil-baths recommended by Wydler‡, from other reasons, must certainly answer the purpose of counteracting the loss of caloric, and deserve the full attention of the profession.

Goltz, in his essay on the influence of the central nervous system (*Virchow's Archiv.*, Vol. 28, p. 428), shows conclusively, that interruption of the muscular tone of a larger portion of the blood-vessels is followed, from purely mechanical causes, by a diminution of the propelling power of the heart; that the blood accumulates rapidly in the relaxed vessels, like it would in a suddenly-established aneurism; that the heart operates laboriously, like a pump without sufficient water; and that if the contractile tone of the vessels ceases, the action of the heart becomes powerless on account of the increased vascular capacity. In extensive burns, this very condition exists, and causes death at once, or after awhile, in connection with the increased loss of caloric.

The sudden death from extensive burns which has been described by many authors as having occurred from inanition, nervous collapse, shock, etc., must be ascribed to such occur-

*Allegem. Wiener Medicinische Zeitung, 1861, No. 43, p. 52.

†Über Athmen nach Unterdrückung der Hautausdünstung. Archiv. für Physiologische Heilkunde, 1858, N. F. 2, p. 488.

‡Archiv. für Klinische Chirurgie, 1865, 6, p. 744.

rences. The comparative absence of palpable lesions in the great viscera in cases of sudden death, as well as the hyperæmic condition in which some of them are found by autopsy of cases of less sudden death, are accounted for by them. The latter, occurring in the brain, air-passages and intestines, are passive congestions from paralysis of the heart, not active fluxionary congestions from interrupted cutaneous action; and their frequent absence in autopsies, in the most violent lesions of that kind, is undoubtedly owing to the large amount of blood which accumulates and remains in the injured cutaneous tissues.

This paralysis, or rather gradual loss of motory power of the heart, is, therefore, one of the greatest dangers threatening severely burned individuals. The judicious but energetic use of exciting and stimulating remedies is, therefore, decidedly indicated, in spite of Beveridge, who looks upon the increased sensation of pain caused by their use as a new danger to life. The normal or increased frequency of the pulse forms no contra-indication to their use.

The principal therapeutical object, however, in the treatment of extensive burns of the skin, as this paralysis of the heart has the dilatation of the blood-vessels throughout the extent of the injured cutis for its primary cause, must consist in the removal of the cause of this dilatation. Dr. Frank has sufficiently demonstrated that the energetic application of ice will not answer this purpose, while ergotine, when injected into the abdominal cavity of frogs, produced a considerable and marked contraction of the capillaries of their swimming membranes, previously enlarged by scalding with hot water. His experiments in relation to the effect of ergotine on scalded rabbits led to definitely favorable results only in two instances. He ascribes this circumstance to the great difficulty of applying to animals the appropriate amount of stimulation to sustain the action of the heart.

He recommends the careful experimental use of this remedy in severe cases of extensive superficial burns, in the form of hypodermic injections.

Falk Dr. ~~Frank~~ finally enters into the pathology of those cases of burns where the effect of a very intense heat has produced, generally, a less extensive and uniform but much deeper and severer lesion. In such cases, the parts are often burned to a brown or black shriveled crust; and while they generally terminate fatally in a few days, under less dangerous clinical appearances, the dilatation of the blood-vessels and diminution of bodily temperature, so prominent in extensive superficial burns, do not form the principal pathological features of this class of cases; but their autopsies generally reveal inflammations of internal organs, particularly serous membranes, and most prominently, pneumonia.

Dr. ~~Frank~~ Folk mentions, further, the observations of Wertheim, Wilkes and Günzburg*, who have often seen pathological changes in the kidneys after severe burns (mostly nephritis), and also the albuminuria frequently observed by Stokvis†, as a frequent occurrence in the last stages of severe burns. Fischer only saw nephritis in extensive superficial burns of the skin, in those cases where an intense head had caused more limited but severe wounds.

In relation to the causes of pneumonia occurring in these lesions, he does not agree with Bird, who bases this connection between pneumonia and burns of the skin on the similarity of their function—that of respiration—because the respiratory action of the skin occupies entirely too unimportant a position in relation to the respiratory action of the lungs, to suppose that a lesion of the first would produce an inflammation of the other by transferred irritation; and leaving out of consideration those pneumonias which are the consequence of direct inhalation of heated vapors, he thinks it more rational to ascribe the simultaneous occurrence of this disease with burns to the fact, that burned individuals are much more susceptible to the atmospheric changes which often produce pneumonia, than uninjured ones. According to his own observations, these inflammations of the lungs favor the hypostatic forms of zymotic diseases—affect the posterior portions of the lungs principally, and exhibit generally, not fibrinous infiltration (red hepatization), but the

*Uber den Tod durch Verbrennungen. Günzburg Zeitschrift für Klin. Medicin., 1850, 1, p. 401.

†S. Rosenstein: Die Path. u Therapic. der Nierenkrankheiter, 1870, p. 173.

different forms of the soft, not granulated and not so distinctly red, but frequently dirty-red, brown or gray infiltrations, which European pathological anatomists generally designate as splenizations.

He accounts for the albuminuria, observed by Stokvis in these cases, by the fact that this condition is frequently observed in diseases where, by a diminution of the motory power of the heart, a mechanical disturbance of the circulation produces hyperæmia of the kidneys, without a closer participation of these organs in the original pathological process*. He does not deny the vicarious functional relation existing between the kidneys and the skin; but from the rare occurrence of nephritis in his test-animals, he concludes that this disease does by no means necessarily follow burns of the skin.

The most rational cause, however, of pneumonia, nephritis Falk and other inflammations, after deep burns, Dr. ~~Frank~~ thinks, has been indicated by the experiments and observations of Schultze†. He found, that when a small quantity of human blood is exposed on a heatable objective-table, or a larger quantity in a water-bath, to a temperature of over 50° Centigrade (122° Fahrenheit), changes of shape and size of the colored blood corpuscles take place, which consist in a separation of smaller and larger globules from the periphery of the blood discs. These observations correspond with those of Wertheim, who noticed in the blood of his burned test-animals—

1. An unusually large number of small globular bodies (0.001 to 0.0004 millimetre), optically and chemically identical with red corpuscles.

2. Numerous blood-cells, evidently engaged in a process of division.

3. An unusually large number of colorless corpuscles, often equal to the number of the red corpuscles.

Falk Dr. ~~Frank~~ himself has often found, in the blood of his test-animals, forms of blood-cells which reminded him vividly of those that are frequently observed in blood which, either within or outside of the organism, has entered into a process of decom-

*Rose's u Wunderlichs Archiv., Vol. 3, 1844, p. 120.

†Archiv. für Mikroskopische Anatomie, 1865.

position—where, while the colorless corpuscles remain intact, the colored ones have already become decomposed, many have disappeared, and a relative increase of the number of the white corpuscles becomes visible, while an absolute one does not really exist.

There can be no doubt, that if the contents of the blood-vessels undergo such changes by the influence of high degrees of heat, the detritus, the decomposed particles within the scalded vessels, must enter the general circulation, and must materially interfere with the normal life of the blood, which, if the injury reaches any extent, must hasten death, or, if less extensive, must, by interrupting the supply of normal blood to the lungs, kidneys and other viscera, give rise to those circulatory and nutritive changes spoken of above as inflammations of these organs. A very appropriate analogy to this process furnishes the poisoning by coal gas (carbonic oxyde gas), where, after the first dangerous symptoms are often successfully overcome, the chemical alteration of the colored corpuscles, initiated by the inspiration of the noxious gas, is often sufficient to establish pneumonia and nephritis long after all the gas has been eliminated from the system. This interruption of the supply of normal blood to these important viscera Dr. ~~Frank~~ Falk considers the cause of the inflammations to which they are so often subject in this class of burns of the skin. That these inflammations exhibit rather an adynamic and hemorrhagic character, can not be wondered at, when they are based upon the decomposition of a large number of red blood corpuscles. Early transfusion of healthy blood is recommended by him in deep burns of the skin, as the principal remedy to prevent these inflammations, or to bring them within the boundaries of successful treatment.

The duodenal ulcerations principally observed by Carling*, and afterward by Rokitansky, as consequences of deep burns, are—after the etiology of ulcerations of the stomach, first established by Virchow—considered by Dr. ~~Frank~~ Falk as the consequences of circulatory disturbances in the capillaries of the mucous membrane, depending upon deficient cardiac action.

*On Acute Ulceration of the Duodenum. Medico-Chirurgie Transactions, Vol. 25, p. 260.

The necrotic condition of the blood in these lesions, spoken of above, and the decomposing influences of the intestinal contents, assist in the completion of the process. In the same manner, we find ulcerations in the stomach and duodenum in other affections, where the cardiac action has been depressed and the morphotic condition of the blood injured—for instance, by the abuse of alcoholic drinks*, or by the effect of very low temperature, as in freezing of the body.

A remarkable physiological fact is contained in the identity of the effect of very low (below 0° Centigrade—32° Fahrenheit) and of very high temperatures (above 50° Centigrade—122° Fahrenheit) on the blood and its corpuscles, first observed by Schultze, and afterward by Pouchet†.

